# Role of Surface Coverage and Film Quality of the TiO_2_ Electron Selective Layer for Optimal Hole-Blocking Properties

**DOI:** 10.1021/acsomega.1c06622

**Published:** 2022-03-31

**Authors:** Syeda Qudsia, Staffan Dahlström, Christian Ahläng, Emil Rosqvist, Mathias Nyman, Jouko Peltonen, Ronald Österbacka, Jan-Henrik Smått

**Affiliations:** † Laboratory of Molecular Science and Engineering, Faculty of Science and Engineering, Åbo Akademi University, Henriksgatan 2, 20500 Turku, Finland; ‡ Physics, Faculty of Science and Engineering, Åbo Akademi University, Henriksgatan 2, 20500 Turku, Finland

## Abstract

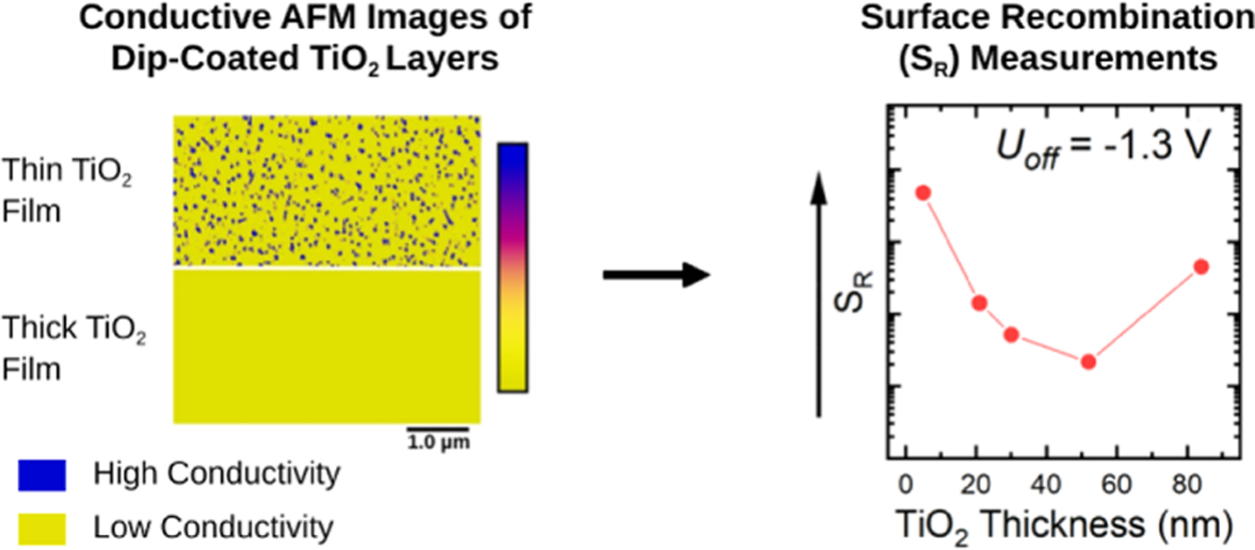

Titanium dioxide (TiO_2_) is a commonly used electron selective layer in thin-film solar cells. The energy levels of TiO_2_ align well with those of most light-absorbing materials and facilitate extracting electrons while blocking the extraction of holes. In a device, this separates charge carriers and reduces recombination. In this study, we have evaluated the hole-blocking behavior of TiO_2_ compact layers using charge extraction by linearly increasing voltage in a metal–insulator–semiconductor structure (MIS-CELIV). This hole-blocking property was characterized as surface recombination velocity (*S*_R_) for holes at the interface between a semiconducting polymer and TiO_2_ layer. TiO_2_ layers of different thicknesses were prepared by sol–gel dip coating on two transparent conductive oxide substrates with different roughnesses. Surface coverage and film quality on both substrates were characterized using X-ray photoelectron spectroscopy and atomic force microscopy, along with its conductive imaging mode. Thicker TiO_2_ coatings provided better surface coverage, leading to reduced *S*_R_, unless the layers were otherwise defective. We found *S*_R_ to be a more sensitive indicator of the overall film quality, as varying *S*_R_ values were still observed among the films that looked similar in their characteristics via other methods.

## Introduction

The emergence of thin-film solar cell technologies that can be processed from solutions offer diverse and cost-effective alternatives for harvesting solar energy. Accompanied by a recent surge in efficiencies,^1,2^ organic and perovskite solar cells are now closer to their commercial realization. These solar cells typically consist of an absorber layer sandwiched between two carrier selective layers: an electron selective layer (ESL) and a hole selective layer (HSL). The ESL extracts electrons and transports them while blocking holes, and the opposite processes take place at the HSL.

Metal oxides are commonly used as carrier selective layers. Among them, TiO_2_ has been used extensively as an ESL in perovskite solar cells,^3,4^ organic and hybrid solar cells,^5−7^ as well as dye-sensitized solar cells.^8^ TiO_2_ is a nontoxic semiconductor that is chemically and optically stable.^9^ From a device point of view, TiO_2_-based layers are cheap and easy to fabricate, have long electron diffusion lengths, and exhibit favorable energy-level alignment with most absorber layers in solar cells.^3,5,8,10,11^ This enables the extraction of electrons by the TiO_2_ layer but prevents holes from leaving the device through it.^3,6^

The properties of these layers are determined primarily by their synthesis process and conditions, and directly affect the performance of fabricated devices. TiO_2_ has been deposited by a variety of techniques, such as spin coating,^3,7,12,13^ dip coating,^14,15^ spray pyrolysis,^7,12,16−18^ chemical bath deposition,^19^ electron beam evaporation,^20^ chemical vapor deposition,^21^ RF sputtering,^22^ and atomic layer deposition,^12,17^ among others. TiO_2_ layers deposited by atomic layer deposition have a reduced number of pinholes and result in high-efficiency devices.^12,17^ Other methods, like dip coating, spin coating, and spray pyrolysis, are cost-effective, but layers deposited by these methods are not immune to defects such as pinholes.^3,7,12,14,15,17^ Dip coating and spray pyrolysis are also attractive techniques as they are simple, versatile, and easy to scale-up.^16,23^

The quality of an ESL can be evaluated in terms of its hole-blocking properties. An ideal contact will efficiently extract all electrons while blocking all holes. However, in a real case, the presence of defects and gap states will serve as recombination centers, leading to recombination across the interface, and will manifest as a recombination current going through the device.^24^ The hole-blocking property of an ESL is also severely affected by structural irregularities, such as pinholes and inadequate surface coverage.^3,4,14,17,25^ A poorly blocking ESL will have increased surface recombination and voltage losses, decreasing the open-circuit voltage and fill factor.^26^ Hence, good-quality contacts are essential for high-efficiency solar cells.

**Scheme 1:**
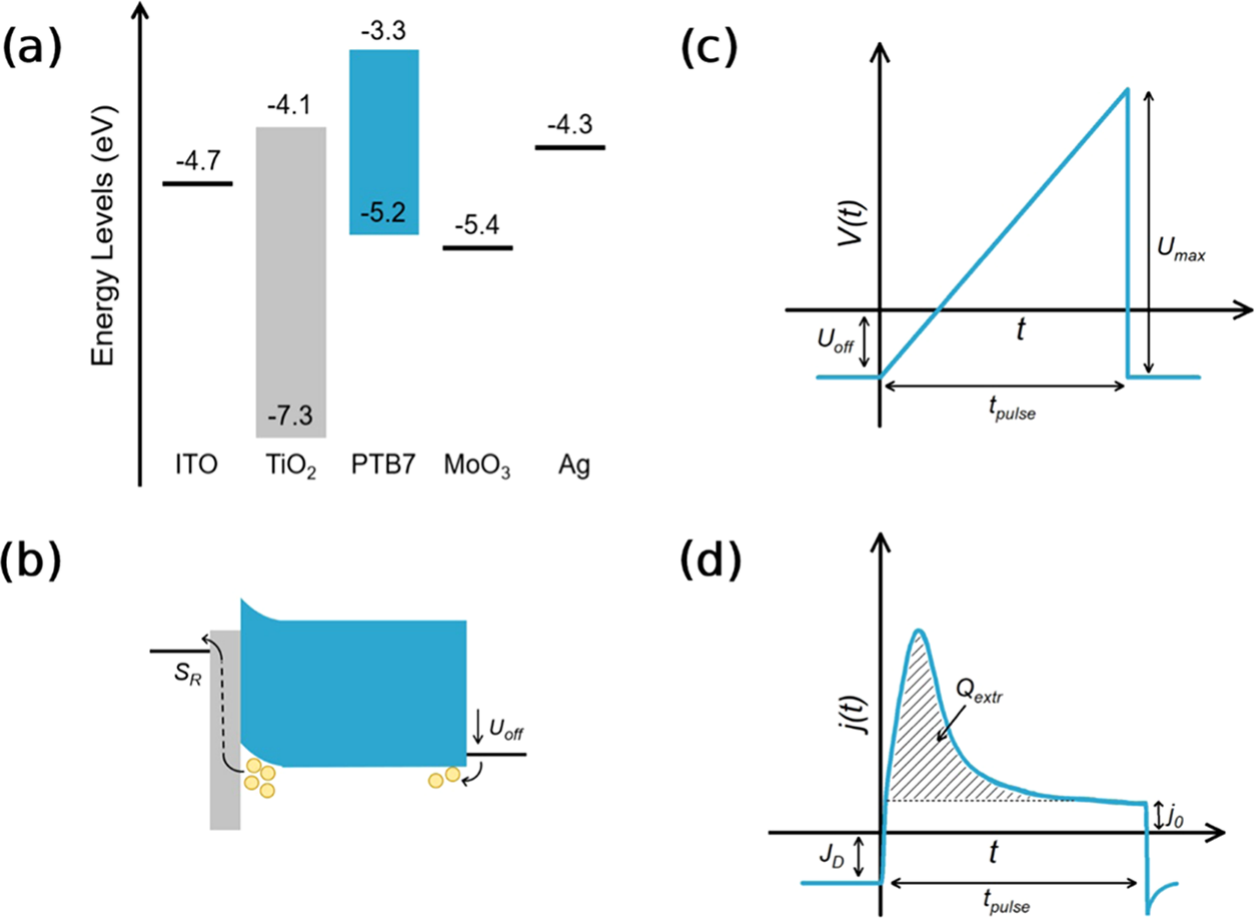
Schematic Illustrations of MIS-CELIV Measurements (a) Energy levels of the materials used in the devices for CELIV measurements. ITO is the bottom electrode, TiO_2_ acts as a hole-blocking layer, PTB7 is the organic semiconductor, and MoO_3_:Ag is the hole-injecting top contact. (b) Application of an offset voltage, *U*_off_, that is greater than the built-in voltage of the device causes injection of holes into the device, some of which recombine with electrons across the TiO_2_/PTB7 interface with some surface recombination velocity, *S*_R_. (c) CELIV pulse: *U*_off_ is the offset voltage applied before the linear voltage pulse, *t*_pulse_ is the pulse length, and *U*_max_ is the maximum voltage of the pulse. (d) Resulting current transient, with *J*_D_ being the steady-state dark current flowing through the device, *j*_0_ is the displacement current given by the geometric capacitance of the device, and *Q*_extr_ refers to the extracted charge.

The hole-blocking properties of an ESL can be determined via charge extraction by linearly increasing voltage (CELIV) in metal–insulator–semiconductor structures (MIS-CELIV)^24,27^ (Scheme 1). For the determination of the hole-blocking behavior of an ESL by MIS-CELIV, a hole-only device is used. The required device incorporates the studied ESL (TiO_2_) in place of the insulator in an MIS structure and a semiconducting polymer (i.e., poly[[4,8-bis[(2-ethylhexyl)oxy]benzo[1,2-*b*:4,5-*b*′]dithiophene-2,6-diyl][3-fluoro-2-[(2-ethylhexyl)carbonyl]thieno[3,4-*b*]thiophenediyl]]; PTB7) as a hole-transporting material. An ohmic contact (MoO_3_:Ag) is employed to inject holes into the device that are blocked upon reaching the ESL due to the lower valence band position for TiO_2_ (−7.3 eV) compared to the HOMO level of PTB7 (−5.2 eV) (Scheme 1a). The lower valence band position of TiO_2_ also prevents the injection of holes from indium tin oxide (ITO) into the device, hence acting as an injection barrier, which is required for the realization of a hole-only device.

During the measurement, a steady-state offset voltage (*U*_off_) is applied in such a way that the injecting contact starts injecting holes when *U*_off_ becomes larger than the built-in voltage (*V*_bi_) of the device (Scheme 1b). These carriers accumulate close to the blocking layer and are subsequently extracted by applying a linearly increasing voltage pulse in reverse bias (Scheme 1c). By integrating the obtained extraction current transient, the extracted charge (*Q*_extr_) can be determined (Scheme 1d). From this, the surface recombination velocity, *S*_R_, for holes can be determined by the following equation^24,27^
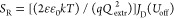
1where εε_*0*_ is the permittivity of the semiconductor layer, *k* is the Boltzmann constant, *T* is the temperature in Kelvin, *q* is the elementary charge, and *J*_D_(*U*_off_) is the steady-state current density in the dark at the applied *U*_off_ > *V*_bi_.

In this work, we have used the hole-blocking properties of the selective layer as a measure of the quality of the deposited compact layer. TiO_2_ layers were fabricated using sol–gel chemistry with dip coating. In dip coating, the thickness of the film can be controlled either by the rate at which the substrates are withdrawn from the solution or by increasing the concentration of the precursor in the sol.^23^ We deposited TiO_2_ layers of different thicknesses by increasing the precursor concentration while keeping the withdrawal rate constant during dip coating. These layers were made on two substrates: indium tin oxide (ITO) and fluorine-doped tin oxide (FTO). For charge extraction measurements, a semiconducting polymer was spin-coated on the TiO_2_ layers, followed by evaporation of the injecting contact. Sensitive microscopic and spectroscopic techniques were used to analyze the morphological and conductive properties and quality of the deposited TiO_2_ films. MIS-CELIV was used to quantify the hole-blocking properties of the deposited layers by calculating the *S*_R_ velocity for holes at the interface between the TiO_2_ and the polymer layers. In this way, we have used MIS-CELIV to investigate the hole-blocking properties of layers fabricated by dip coating and how different thicknesses and substrate roughness impact the quality of the TiO_2_ electron selective layer. As MIS-CELIV was done on 4–6 mm^2^ device areas, the resulting *S*_R_ values give a better idea of the optimal thickness of TiO_2_ needed to fully cover the substrate compared to other more traditional surface characterization techniques, such as atomic force microscopy (AFM) and X-ray photoelectron spectroscopy (XPS).

## Results and Discussion

In this work, different thicknesses of TiO_2_ layers were prepared by dip coating. By increasing the molar ratio of TiCl_4_ in the solution, we obtained TiO_2_ layers of 5, 21, 30, 52, and 84 nm thickness, as confirmed with X-ray reflectometry (XRR) (Table 1). Glass substrates were preferred for measurements with XRR, as the roughness of conductive glass substrates makes it difficult to determine the thickness with this technique. A decrease in density can be seen with increasing thickness, likely caused by shrinkage during annealing.^15^ The lowest density is observed for the 84 nm TiO_2_ film, as thicker layers are more prone to lateral tensile stresses during film densification and annealing, leading to the formation of cracks and pinholes in the final layer^14,23^ (Figure S1 in the Supporting Information).

**Table 1:** Thickness and Density of Dip-Coated TiO_2_ Layers (on Glass Substrates), as Measured by XRR^a^

TiCl_4_ molar ratio (mol)	TiO_2_ thickness (nm)	TiO_2_ density (g/cm^3^)
1	5	3.51
3	21	3.48
5	30	3.44
7	52	3.29
10	84	3.15

a The standard deviations in thickness and density between three films of the TiCl_4_ molar ratio are typically below 4%.

Dip-coated layers were deposited on two conductive glass substrates: indium tin oxide (ITO) and fluorine-doped tin oxide (FTO). A reduction in sample roughness is seen after coating the substrates with different thicknesses of TiO_2_ (Table 2), as the topographical valleys of the substrate are gradually filled up with increased amounts of deposited TiO_2_.^15^ As the ITO substrate is smoother than FTO, a thinner TiO_2_ layer is presumably needed for good surface coverage.

**Table 2:** Roughness Parameters for the Bare ITO and FTO Substrates, and Those with an Increasing Thickness of the TiO_2_ Layer (Determined from 5 μm × 5 μm AFM Images with a Resolution of 512 × 512 Pixels)^a^

TiO_2_	ITO			FTO		
	*S*_a_ (nm)	*S*_q_ (nm)	*S*_dr_ (%)	*S*_a_ (nm)	*S*_q_ (nm)	*S*_dr_ (%)
0 nm	2.9 ± 0.1	3.6 ± 0.1	2.2 ± 0.1	13.2 ± 0.2	16.5 ± 0.3	26.0 ± 1.0
5 nm	2.24 ± 0.02	2.95 ± 0.02	1.5 ± 0.1	12.0 ± 0.1	15.0 ± 0.2	21.0 ± 2.0
21 nm	1.66 ± 0.03	2.06 ± 0.03	0.4 ± 0.1	9.9 ± 0.1	12.3 ± 0.1	9.6 ± 0.1
30 nm	1.56 ± 0.01	2.00 ± 0.03	0.34 ± 0.02	8.5 ± 0.3	10.6 ± 0.5	6.2 ± 0.2
52 nm	1.20 ± 0.02	1.48 ± 0.02	0.08 ± 0.00	5.9 ± 0.2	7.5 ± 0.2	1.67 ± 0.01
84 nm	3.0 ± 1.0	5.0 ± 1.0	0.4 ± 0.2	3.13 ± 0.03	4.01 ± 0.04	0.27 ± 0.01

a *S*_a_ is the average height deviation from the arithmetic mean, *S*_q_ is the root-mean-square roughness, and *S*_dr_ is the surface area ratio. Standard deviations are also included.

**Figure 1:**
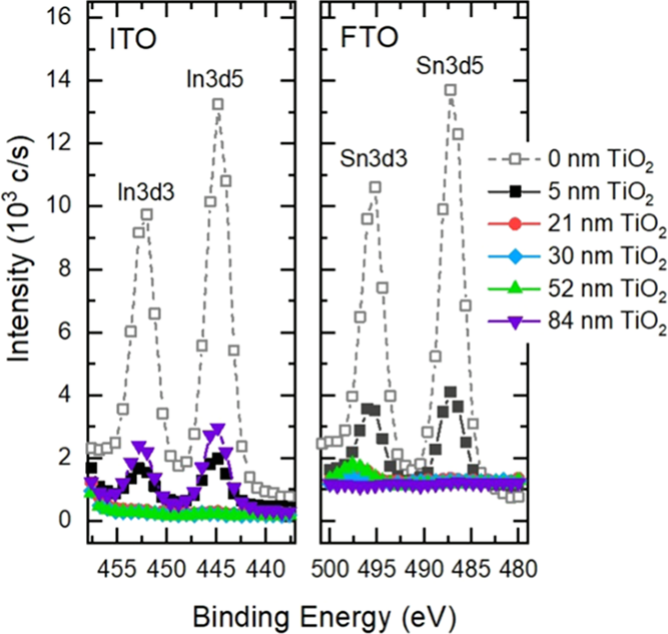
X-ray photoelectron spectroscopy of bare and coated substrates. The signal of indium, In, from ITO substrate is shown (left) along with the signal from tin, Sn (right), in the case of FTO substrates.

The degree of surface coverage was estimated using XPS (Figure 1). XPS is a surface-sensitive technique and gives elemental information of about 5–10 nm of sample depth. For ITO, indium, In, was chosen as the reference element. ITO substrates without any coating give a strong In signal. The area under the In3d5 peak was normalized to 100%, and TiO_2_-covered substrates were compared to this. For the 5 nm TiO_2_ sample, the intensity is reduced to about 12.5% and further down below 1% for thicker layers. Lower signal intensity from the underlying substrate was attributed to surface coverage with TiO_2_ films of adequate thickness. For the 84 nm TiO_2_ sample, the peak intensity again increases up to 22%.

In the case of FTO, tin, Sn, was probed as the reference element. The intensity of the Sn3d5 peak from the bare substrate was normalized to 100%, similar to ITO. For the 5 nm TiO_2_ sample, however, the intensity appears much higher than the same thickness on ITO, with an intensity of about 24% of Sn3d5 peak in the spectrum of bare FTO. This is reduced below 1% for thicker layers. A higher signal from the substrate in the case of 5 nm TiO_2_ on FTO indicates incomplete surface coverage or TiO_2_ films being extremely thin. Also, the 84 nm TiO_2_ layer appears to behave differently in FTO and no decrease in surface coverage is seen in the XPS data. The roughness of the substrate likely provides some mechanical support to hold the thick TiO_2_ layer more intact compared to the smoother ITO substrate. On ITO, the thick layer is prone to forming structural defects because of lateral tensile stress during the annealing process^14,23^ (Figure S1 in the Supporting Information), and the exposed substrate contributes to the In3d5 signal in the XPS data in Figure 1. No signals from chlorine were observed in the XPS data, indicating that the TiCl_4_ precursor has been fully converted to TiO_2_ in the deposition and sintering processes.

**Figure 2:**
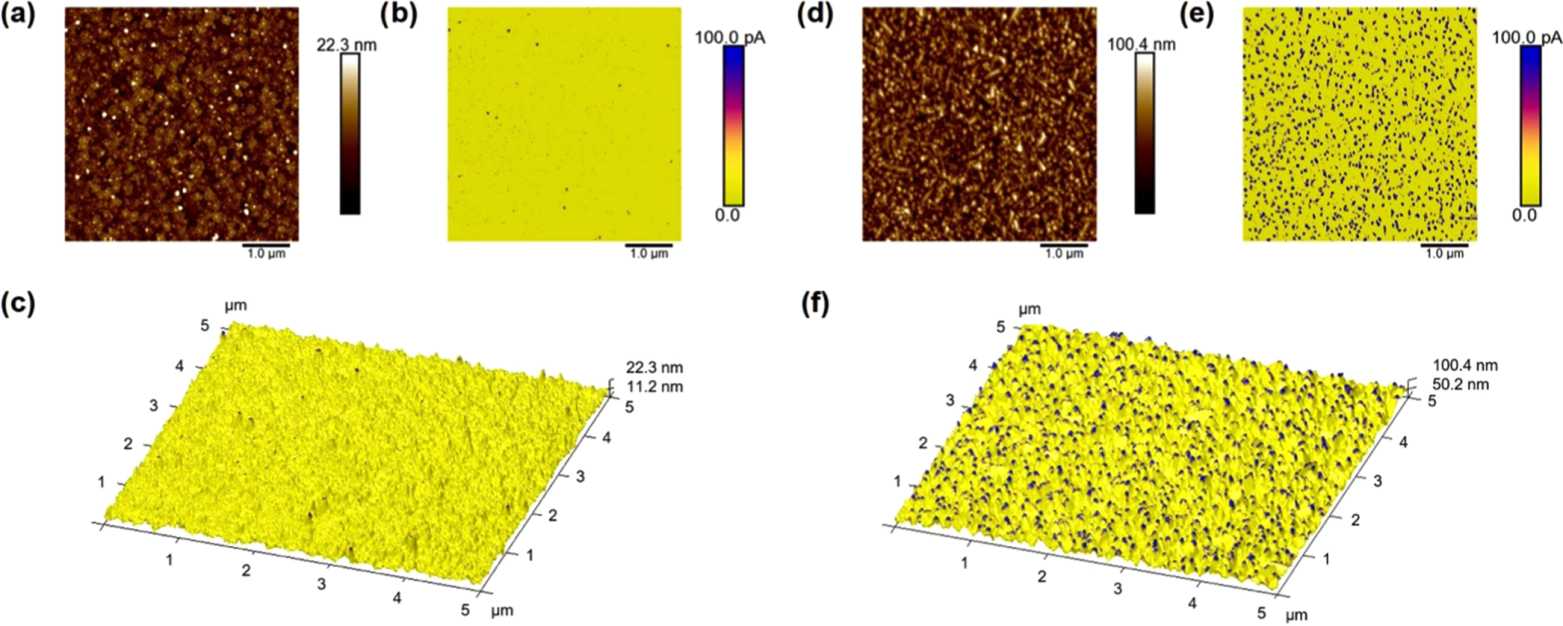
AFM and conductive atomic force microscopy (CAFM) images (5 μm × 5 μm) of 5 nm TiO_2_ on ITO and FTO. (a–c) AFM images of TiO_2_ deposited on ITO; (a) AFM height image, (b) CAFM image with the tip-sample currents on exactly the same spot as in image (a). (c) Three-dimensional (3D) AFM height image overlayed with the color scale of the CAFM image. (d–f) AFM images of 5 nm TiO_2_ deposited on FTO; (d) height image and (e) CAFM image. (f) 3D AFM height image overlayed with the color scale of the CAFM image.

The TiO_2_-coated samples were imaged with both conventional AFM (topography) and CAFM to visualize the level and local differences of conductivity of the TiO_2_ coatings (Figure 2). When the tip-sample bias voltage is below the breakdown voltage, the substrate areas covered with a TiO_2_ film should exhibit low tip-surface currents, whereas currents through the uncoated substrate are expected to remain high.^28^ For bare ITO and FTO, over 95% of the surface area appears conductive with measured currents over 100 pA. Nonconductive areas of the bare substrates without any TiO_2_ coating could be due to inadequate tip-sample contact due to local surface contamination.

On ITO, even with as thin as a 5 nm TiO_2_ coating, the major part of the coated area is found to be nonconductive (current below 100 pA at 1 V bias) and only less than 0.13% of the imaged area appears conductive (current above 100 pA) (Figure 2a–c). On FTO, for the same TiO_2_ thickness, about 8% of the imaged surface was conductive (Figure 2d–f). The conductive fraction of the surface is interpreted to represent a surface with an incomplete TiO_2_ coating, i.e., a coating consisting of pinholes. As the thickness of the TiO_2_ layer increases, the fraction of uncoated regions decreases, i.e., the fraction of the conductive area of the coated surface decreases (Figures S2 and S3 in the Supporting Information). This is supported by the corresponding height images, which were measured simultaneously, where increasing TiO_2_ thickness results in a smoother surface (Table 2). This indicates that the thicker TiO_2_ coatings fill up the valleys of the substrate more effectively. From the relatively high In3d5 peak in the XPS results of the 84 nm TiO_2_ on the ITO sample, one would expect some highly conductive areas in the CAFM measurements. While this was not observed in the small-scale 5 μm × 5 μm CAFM image (Figure S2f), we were able to observe some bare patches in the larger 20 μm × 20 μm image of the same sample (Figure S1 and insets in Figure S2f). As the XPS sampling area is larger still (100 μm), it is likely that we are observing (at least partially) one of these bare patches, which contribute to the increased In3d5 signal in XPS. Average roughness, *S*_a_, root-mean-square roughness, *S*_q_, and surface area ratio, *S*_dr_, for the different coatings and the uncoated substrates are shown in Table 2. The values of all three roughness parameters decrease as the thickness of the TiO_2_ coating increases, both for the ITO and the FTO substrates. However, for the thickest TiO_2_ coating, 84 nm, the roughness parameters increase in the smoother ITO substrate. The increase is caused by structural defects formed in the TiO_2_ layer due to shrinkage during the annealing process. The influence of the underlying roughness on the coating can be seen by comparing the sample series on both substrates. The uncoated FTO glass, with a 5-fold *S*_a_ compared to the uncoated ITO, results in the coating roughnesses being higher throughout the entire series; 5-fold for *S*_q_ and between 10- to 20-fold for *S*_dr_.

The prepared layers on both substrates were processed into devices for charge extraction measurements. For the semiconducting layer, PTB7 was spin-coated on top of the TiO_2_ films. The thickness of PTB7 was approximately 500 nm, determined by AFM (data not shown). A MoO_3_:Ag contact was evaporated on top of the PTB7 layer as the hole-injecting contact. A steady-state *U*_off_ > *V*_bi_ was applied to inject holes into the device that were expected to be blocked at the PTB7/TiO_2_ interface (Scheme 1b,c).

**Figure 3:**
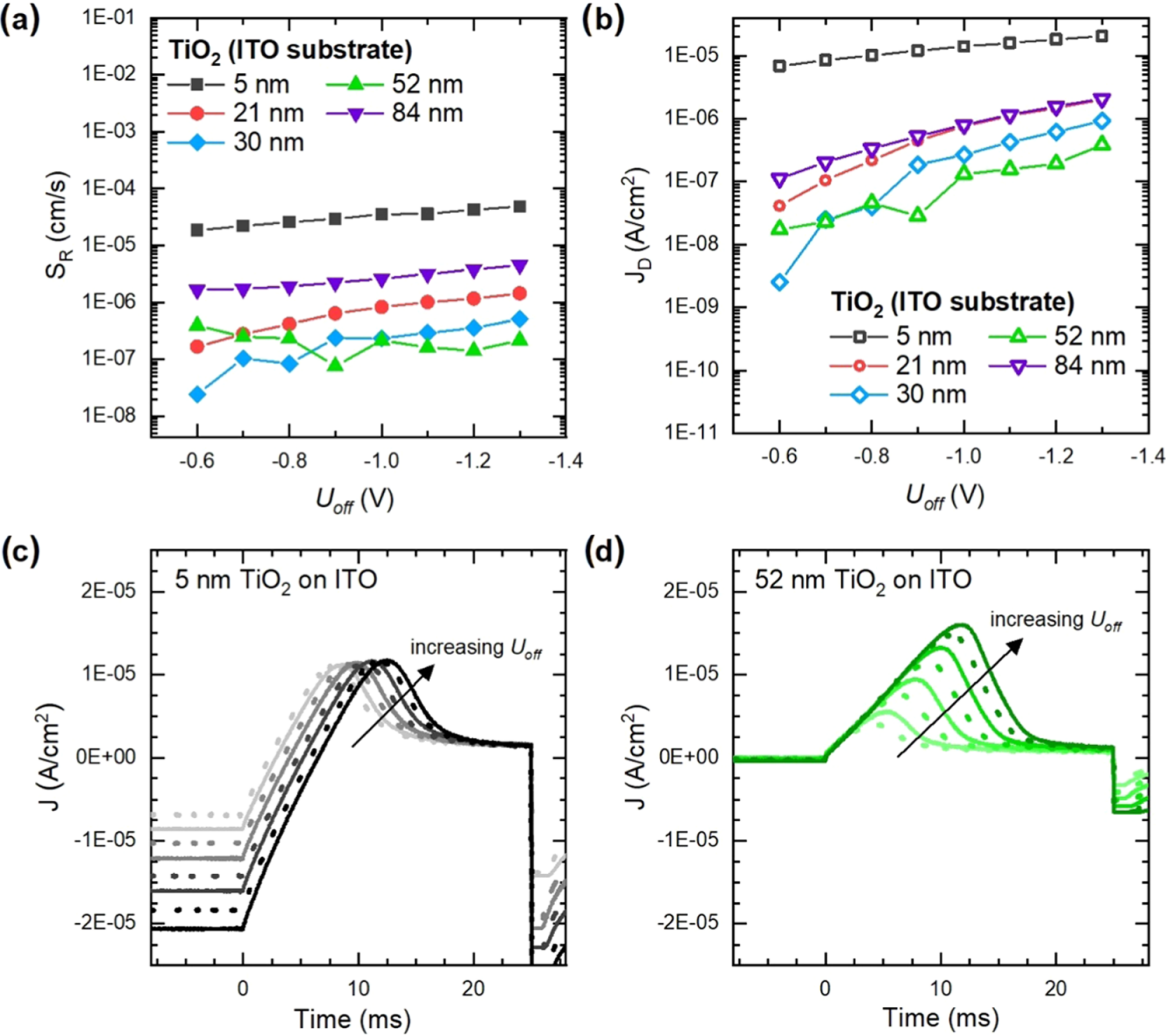
Information from MIS-CELIV measurements. (a) Surface recombination velocities (*S*_R_) for different thicknesses of TiO_2_. (b) Steady-state dark current (*J*_D_) as a function of applied offset voltage (*U*_off_) for the TiO_2_ layers deposited on ITO substrate. (c, d) CELIV transients for 5 and 52 nm TiO_2_ on ITO, respectively, from −0.6 to −1.3 V *U*_off_.

Calculated surface recombination velocity (*S*_R_) values for different thicknesses of TiO_2_ on the ITO substrate are shown in Figure 3a. A decrease in *S*_R_ is seen with increasing TiO_2_ layer thickness. The lowest *S*_R_ values can be observed for 30 nm and 52 nm thick TiO_2_ layers. We expect that the layers are thick enough to cover the substrate and have a low number of pinholes for these thicknesses. For the 84 nm TiO_2_ layer, where we can see morphological defects due to shrinkage during thermal annealing, *S*_R_ values increase again. In this way, *S*_R_ can be used as a very sensitive indicator of layer quality and substrate coverage. Furthermore, it should be emphasized that the MIS-CELIV technique provides structural information on a much larger sample area compared to XPS and CAFM (i.e., 4–6 mm^2^), which is more relevant for the overall performance of real devices.

The devices made on FTO showed poor reproducibility, and less than 50% of the devices were viable during measurements (data not shown). On ITO, there is a pronounced increase in the number of viable devices, with over 90% of contacts working, except for the 84 nm TiO_2_ layer, where about 50% of the contacts could be measured. The measurements require that the blocking contact can provide a surface recombination velocity lower than the effective transport velocity for holes injected from the MoO_3_:Ag anode and transported through the semiconductor layer to the interface, i.e., *S*_R_ < μ*kT*/*qd*, where μ is the mobility and *d* is the thickness of the semiconductor layer.^24^ Hence, for the devices that could not be measured, the TiO_2_ layer is not blocking enough. Thinner layers are also prone to breakdown with increasing voltage, while layers that are more uniformly thick are resistant to such behavior.^28^ The poor reproducibility for FTO devices indicates that while the TiO_2_ layers can have a good surface coverage locally (as observed from the CAFM data), sections with more structural defects might be included when the probing area is larger (as in the case of the *S*_R_ values). On ITO, even the thinnest TiO_2_ coatings (5 nm) are blocking enough, albeit with higher values for *S*_R_. No hole-blocking behavior is seen in devices without a TiO_2_ layer (Figure S4 in the Supporting Information).

In the case of FTO, while the thinner layers might be qualitatively adequate and have the desired compactness, their performance can fall short due to pinholes in the layer caused by poor coverage of the rougher FTO substrate.^4^ We expect that issues related to incomplete surface coverage can be reduced by using a smoother substrate, like ITO. Thus, the roughness of the substrate plays a major role in the final performance of the devices. The resistivity of ITO increases upon annealing at temperatures 400 °C and above,^29,30^ but we did not see much difference for the purpose of conducting CELIV measurements (Figure S4 in the Supporting Information).

Figure 3b shows the steady-state dark current, *J*_D_, measured for devices made on ITO. *J*_D_ is more prominent for thinner films and decreases as the film thickness increases. Figure 3c,d shows transients for the two extreme cases: 5 and 52 nm. *J*_D_ increases prominently for the 5 nm TiO_2_ film with increasing *U*_off_. This could be from contributions of shunt currents through the thin TiO_2_ layer. With the 30 and 52 nm layers, a very low *J*_D_ is observed, with the lowest *J*_D_ seen in 52 nm TiO_2_ film (Figure 3b,d). Meanwhile, the hole reservoir is apparent in the extraction current transients for all sample thicknesses, indicating that some hole-blocking properties are also present for the thinnest films (Figure 3c). From the MIS-CELIV data, it can be seen that minute surface defects are still present in layers with increasing thickness and contributing to *S*_R_, although surface coverage appears to be adequate for these in the data from XPS and CAFM.

## Conclusions

In this work, we clarify the impact of the thickness of the dip-coated compact TiO_2_ layers and the effect of substrate roughness on the hole-blocking properties of such layers was investigated. Complete surface coverage by a compact layer is essential to ensure good blocking properties for minority charge carriers at the extracting interface. As smoother substrates are easier to cover, relatively thin compact layers can provide a good surface coverage. However, rougher substrates such as FTO require thicker TiO_2_ layers. A rougher base substrate results in a higher roughness even for relatively thick coatings. Besides surface coverage, the quality and homogeneity of the coating are essential for its functionality. The conductive imaging mode of AFM, CAFM, proved to be a powerful tool for studying the conductive properties and appearance of pinholes in the TiO_2_ coatings on a micrometer length scale. However, we have demonstrated that MIS-CELIV is an excellent technique to determine the overall quality of the entire compact selective layers and enables us to quantify their hole-blocking properties.

## Experimental Section

ITO (10 ohms/sq, CEC010S, Präzisions Glas and Optik GmbH) and FTO (15 ohms/sq, TEC15, Greatcell Solar) substrates were etched using zinc powder and 2 M HCl solution. They were subsequently washed by sonication in 2% Hellmanex III for 20 min, followed by distilled water (10 + 10 min), acetone (20 min), and isopropanol (20 min). TiO_2_ layers were deposited using dip-coating sols based on TiCl_4_.

A stock solution of TiCl_4_ (>99%, Fluka, Germany) in ethanol (>99.5%, ALTIA Plc, Finland) was prepared, keeping the molar ratio between TiCl_4_ and ethanol as 1:5. For the dip-coating sols, a base solution was prepared in which ethanol, H_2_O, THF (99%, abcr, GmBH), and Pluronic F127 (Sigma-Aldrich) were mixed such that the molar ratio of TiCl_4_/ethanol/H_2_O/THF/Pluronic F127 would remain *x*:250:9.8:20:0.001 in the final solutions (Table 3). An increasing concentration of the TiCl_4_/ethanol stock solution was added to this base mixture. To get a final thickness of 5 nm of TiO_2_, the amount of TiCl_4_/ethanol stock solution was added such that the TiCl_4_ molar ratio (*x*) would be 1 in the final solution. For a 21 nm thickness, the amount of TiCl_4_/ethanol solution was increased such that *x* would be equal to 3. For 30 nm thickness, *x* was kept 5. For 52 nm thickness, *x* was 7, and for 84 nm thickness of the final layer, *x* was equal to 10. The amount of ethanol in the base solutions was adjusted to compensate for ethanol being added from the TiCl_4_/ethanol stock solution.

**Table 3:** Molar Ratio between TiCl_4_/Ethanol/H_2_O/THF/Pluronic F127 with Increasing TiCl_4_ Concentration for Thickness Control of the Final TiO_2_ Layer

TiCl_4_ (mol)	ethanol (mol)	H_2_O (mol)	THF (mol)	Pluronic F127 (mol)
1	250	9.8	20	0.001
3	250	9.8	20	0.001
5	250	9.8	20	0.001
7	250	9.8	20	0.001
10	250	9.8	20	0.001

Immediately prior to TiO_2_ deposition, the substrates were cleaned using air plasma (Harrick Plasma) for 5 min at medium power. Dip coating was done with a withdrawal speed of 85 mm/min. The relative humidity was maintained between 12 and 25% during the dip coating process. The coatings were kept within the dip coating chamber for 8–10 min and dried at 150 °C afterward for about 15 min. They were finally sintered at 500 °C for 30 min.

Devices for CELIV measurements used a semiconducting layer of poly[[4,8-bis[(2-ethylhexyl)oxy]benzo[1,2-b:4,5-b′]dithiophene-2,6-diyl][3-fluoro-2-[(2-ethylhexyl)carbonyl]thieno[3,4-*b*]thiophenediyl]] (PTB7) (Ossila, batch no. M216, MW = 78,852 g/mol). PTB7 solution (80 mg/mL) in chlorobenzene (Sigma-Aldrich) was spin-coated on the prepared substrates with a speed of 700 rpm for 1 min. The films were annealed at 120 °C for 15 min. For the top contact, 10 nm of MoO_3_ was evaporated followed by 60 nm of Ag using a thermal evaporator. Spin coating and evaporation were carried out inside a nitrogen-filled glovebox.

Samples were kept under vacuum (about 10^–5^ mbar) at room temperature for MIS-CELIV measurements. Linearly increasing voltage pulse was generated using a pulse generator (Stanford Research Systems, Inc., model DG 535) and a function generator (Stanford Research Systems, Inc., model DS345), and the response was recorded using an oscilloscope (Keysight, InfiniiVision DSOX3104T). A LabVIEW program was used to control the measurements. A maximum voltage of 4 V was applied for a 25 ms pulse duration. The offset voltage, *U*_off_, was varied between −0.6 and −1.3 V.

The films were prepared on microscope glass slides to determine the thickness of the dip-coated TiO_2_ layers by X-ray reflectometry (XRR). Glass slides were washed with ethanol and treated with air plasma (Harrick Plasma) at medium power for 5 min immediately before dip coating. Dip coating was performed with the same parameters as done on conductive glass substrates. XRR was performed using a Bruker AXS D8 Discover instrument. 2Theta/Omega scanning was done in the range 0.3–3°. Data were analyzed using the LEPTOS software (version 7.03).

For probing the surface coverage, X-ray photoelectron spectroscopy (XPS) was performed using a Physical Electronics, Quantum 2000 instrument. Survey scanning was performed with a monochromatic Al Kα source and a pass energy of 187.85 eV, with a beam diameter of 100 μm. The spectra were analyzed with the MultiPak software (version 9.8.0.19). FWHM/area functionality was used in the MultiPak software for area under the curve analyses.

Atomic force microscopy (AFM) along with its added imaging mode, conductive atomic force microscopy (CAFM) was carried out with a MultiMode 8 instrument from Bruker. Antimony-doped silicon tips with a PtIr coating (SCM-PIC-V2, Bruker) were used. The tip radius was about 25 nm with a spring constant of approximately 0.1 N/m, as reported by the manufacturer. Deflection sensitivities of the cantilevers ranged between 102–145 nm/V, and the images were scanned with a force of ∼5 nN. Before scanning, the samples were cleaned using a plasma cleaner for 5 min at medium power; 5 by 5 μm images (resolution 512 by 512 pixels) were obtained at a scan speed of 1 Hz. A bias of 1 V was applied. The images were analyzed using Nanoscope Analysis software (version 2). The captured images were flattened using first-order flattening. A 100 pA cutoff was used for the current scale.

## Supporting Information Available

Images from optical microscopy and AFM of 84 nm TiO_2_ film on ITO; AFM images of all samples done on ITO; AFM images of all samples done on FTO; CELIV transients for ITO/PTB7/MoO_3_:Ag devices without TiO_2_ blocking layer, with ITO treated at two different temperatures (PDF)
