# Supplementary material for: Role of Surface Coverage and Film Quality of the TiO2 Electron Selective Layer for Optimal Hole-Blocking Properties
Source: ACS Omega. 2022 Mar 31;7(14):11688–95. doi: 10.1021/acsomega.1c06622 (PMC9017100; doi:10.1021/acsomega.1c06622)
Supplement: Supplementary file 1 — ao1c06622_si_001.pdf [file ao1c06622_si_001.pdf]

Supporting Information

Role of Surface Coverage and Film Quality of the TiO_2 Electron Selective Layer for Optimal Hole Blocking Properties

Syeda Qudsia [a], *Staffan Dahlström* [b], *Christian Ahläng* [b], *Emil Rosqvist* [a], *Mathias Nyman* [b], *Jouko Peltonen* [a], *Ronald Österbacka* [b], *Jan-Henrik Smått* [a]*

[a] Laboratory of Molecular Science and Engineering, Faculty of Science and Engineering, Åbo Akademi University, Henriksgatan 2, 20500 Turku, Finland

[b] Physics, Faculty of Science and Engineering, Åbo Akademi University, Henriksgatan 2, 20500 Turku, Finland

*Email: jan-henrik.smatt@abo.fi

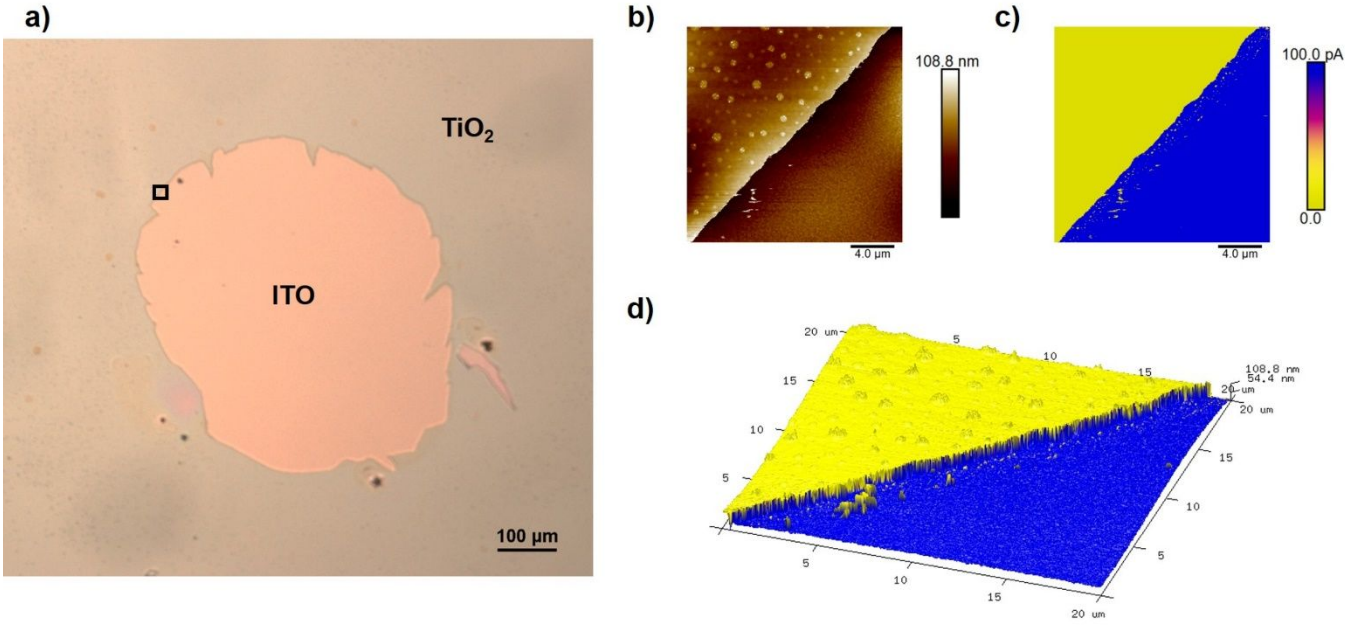

Figure S1. Images for 84 nm TiO_2 on ITO. Part a) shows an optical microscopy image with exposed substrate where the TiO_2 coating has come off. Parts b), c) and d) show 20 x 20 μm^2 AFM image. b) is the height image, c) is the current image from the same area shown in a), and d) is the current map overlayed on the height topograph (height scale).

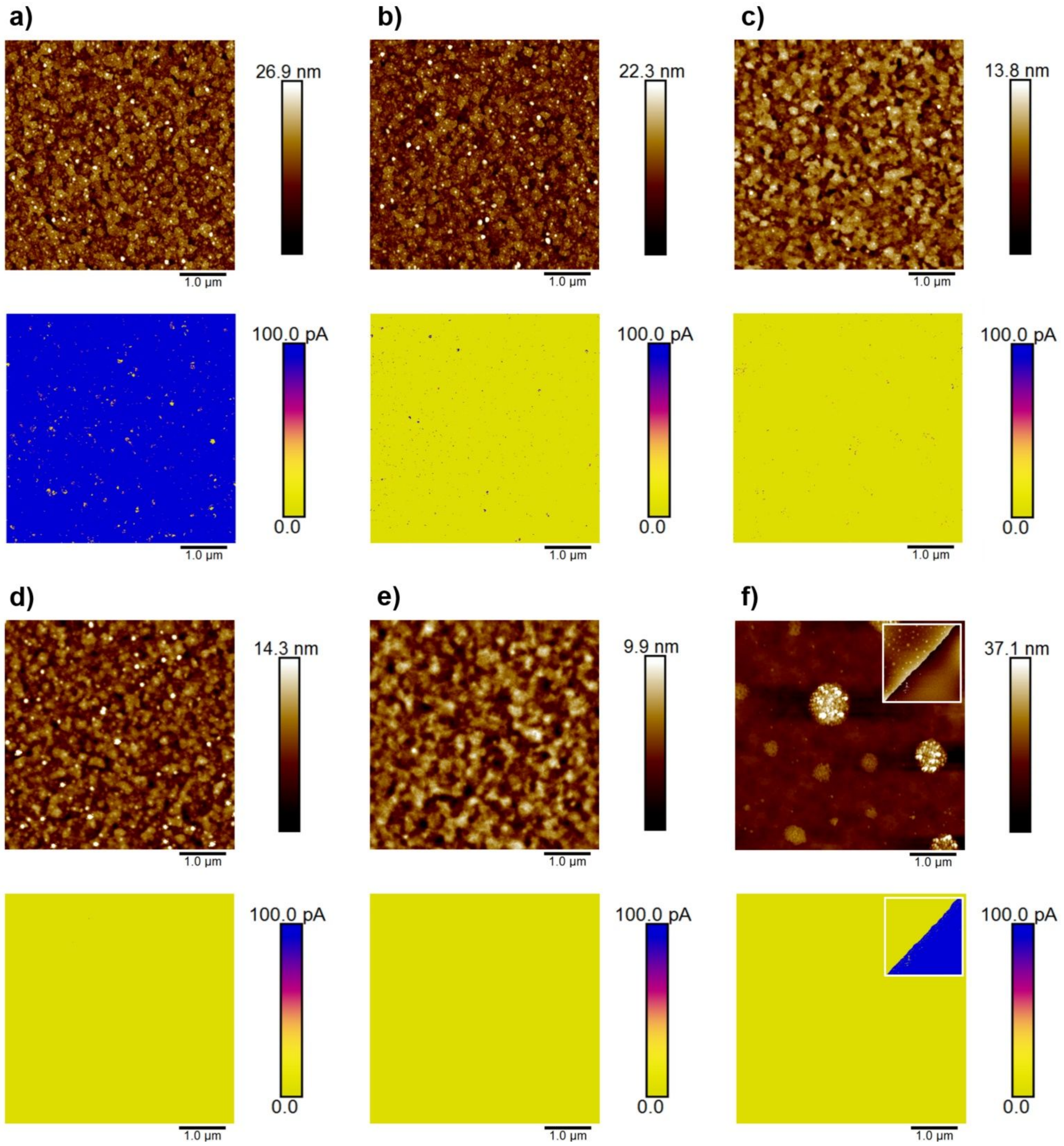

Figure S2. Conductive AFM images (5 µm × 5 µm) of TiO_2 on ITO substrate. AFM height topographs (upper) and current maps (lower) for a) bare ITO, and with a TiO_2 coating on ITO of

b) 5 nm, c) 21 nm, d) 30 nm, e) 52 nm, and f) 84 nm thickness. In f), insets show 20 μm x 20 μm AFM images of exposed ITO area after the TiO_2 coating came off (see Figure S1 for details).

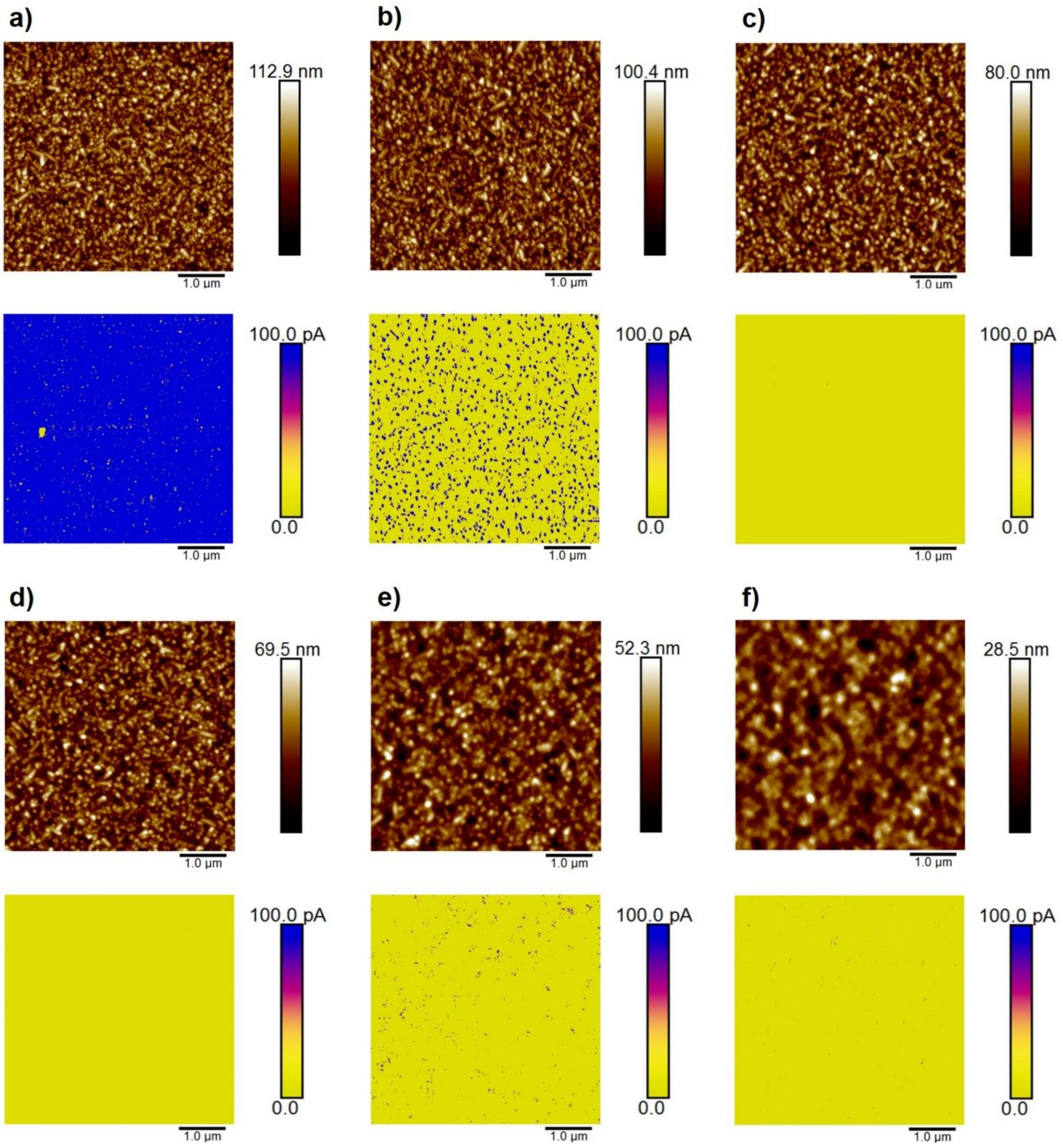

Figure S3. Conductive AFM (CAFM) Images (5 µm × 5 µm) of TiO_2 on FTO substrate. AFM height topographs (upper) and current maps (lower) images for a) bare FTO, and with a TiO_2 coating on FTO of b) 5 nm, c) 21 nm, d) 30 nm, e) 52 nm, and f) 84 nm thickness.

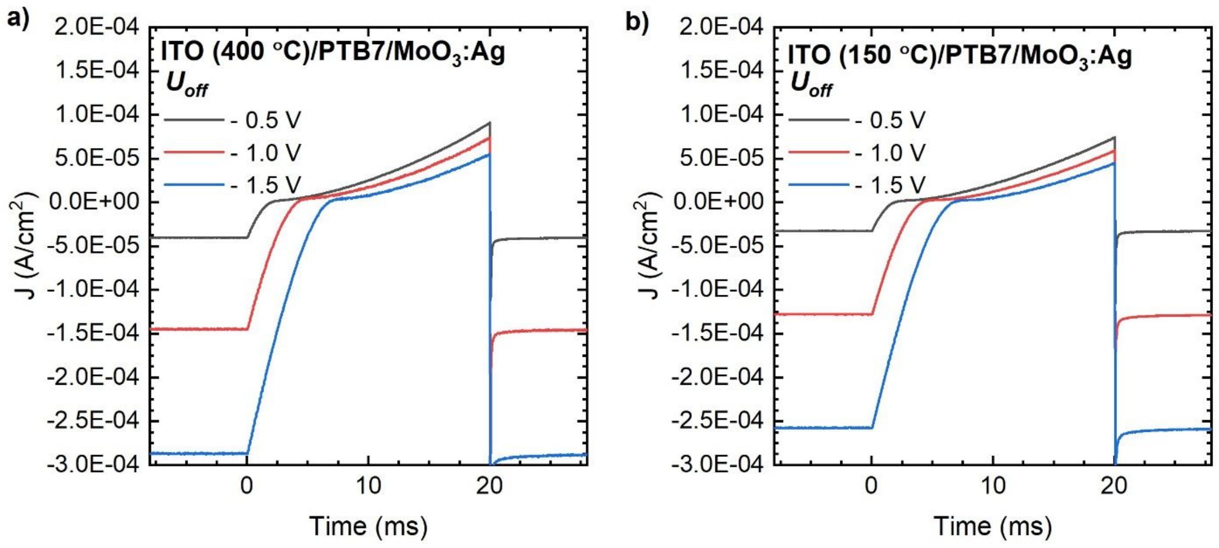

Figure S4. CELIV transients for devices without a TiO_2 blocking layer, with the architecture ITO/PTB7/MoO_3:Ag. ITO has been treated at a) 400 ˚C and b) 150 ˚C.
